# Differential gene expression in migratory streams of cortical interneurons

**DOI:** 10.1111/j.1460-9568.2011.07896.x

**Published:** 2011-11

**Authors:** Mary Antypa, Clare Faux, Gregor Eichele, John G Parnavelas, William D Andrews

**Affiliations:** 1 Department of Cell and Developmental Biology, University College LondonGower Street, London WC1E 6BT, UK; 2 Centre for Neuroscience, University of MelbourneAustralia; 3 Department of Genes and Behaviour, Max Planck Institute for Biophysical ChemistryGöttingen, Germany

**Keywords:** developing cerebral cortex, interneurons, microarray, migration

## Abstract

Cortical interneurons originate in the ganglionic eminences of the subpallium and migrate into the cortex in well-defined tangential streams. At the start of corticogenesis, two streams of migrating neurons are evident: a superficial one at the level of the preplate (PPL), and a deeper one at the level of the intermediate zone (IZ). Currently, little is known about the signalling mechanisms that regulate interneuron migration, and almost nothing is known about the molecules that may be involved in their choice of migratory stream. Here, we performed a microarray analysis, comparing the changes in gene expression between cells migrating in the PPL and those migrating in the IZ at embryonic day 13.5. This analysis identified genes, many of them novel, that were upregulated in one of the two streams. Moreover, polymerase chain reaction, *in situ* hybridization experiments and immunohistochemistry showed the expression of these genes in interneurons migrating within the PPL or IZ, suggesting that they play a role in their migration and choice of stream.

## Introduction

Interneurons, which constitute a morphologically, neurochemically and functionally diverse group of cortical cell types, are essential modulators of neuronal activity in the cerebral cortex. Abundant evidence indicates that alterations in the number, distribution and function of these GABA-releasing inhibitory neurons in humans may lead to neurological and psychiatric disorders (Benes & Berretta, 2001; Cossart *et al.*, 2005; Gant *et al.*, 2009). Thus, the mechanisms that control interneuron development have received considerable attention in recent years.

Tracing, fate-mapping and loss of function analyses in rodents have shown that the vast majority of cortical interneurons arise from the medial ganglionic eminence (MGE) and caudal ganglionic eminence (Tamamaki *et al.*, 1997; Lavdas *et al.*, 1999; Nery *et al.*, 2002; Xu *et al.*, 2004) and from the embryonic preoptic area (Gelman *et al.*, 2009). These studies have also traced in detail the three tortuous migratory paths that interneurons follow from their origins in the subpallium to the cortex (reviewed by Corbin *et al.*, 2001; Marín & Rubenstein, 2003; Métin *et al.*, 2006). Specifically, an early cohort [embryonic day (E)12 in the mouse] first reaches the cortex and migrates at the level of the preplate (PPL). Slightly later in development (E13–15), a second and more prominent cohort migrates predominantly through the intermediate zone (IZ). At the later stages of corticogenesis and after the formation of the cortical plate (CP), three distinct tangential migratory streams are in evidence in the developing cortex, located in the marginal zone (MZ), lower IZ/subventricular zone (SVZ), and subplate (SP) (Lavdas *et al.*, 1999; Anderson *et al.*, 2001; Métin *et al.*, 2006). Intricate molecular mechanisms are at play in the subpallium, repelling interneurons from their origins and sorting them into striatal or cortical types. The molecules involved in these processes include the Slit proteins and their Robo receptors, as well as the class 3 semaphorin family and their neuropilin and plexin receptors (Marín *et al.*, 2001; Andrews *et al.*, 2006; Barber *et al.*, 2009; Hernández-Miranda *et al.*, 2011). Migrating interneurons destined for the cortex utilize attractive cues, prominent among them neuregulin1/ErbB4 (Flames *et al.*, 2004), to move round the corticostriatal notch and enter the cortical mantle (reviewed by Hernández-Miranda *et al.*, 2010).

A number of factors have been identified as regulators of tangential (and radial) interneuron migration, including neurotrophic factors (brain-derived neurotrophic factor, neurotrophin 4, and glial cell-derived neurotrophic factor) (Polleux *et al.*, 2002; Pozas & Ibáñez, 2005) and the chemokine CXCL12 (Stumm *et al.*, 2003; Liapi *et al.*, 2008). CXCL12 is produced by meningeal cells, Cajal–Retzius (CR) cells in the MZ, and cells in the IZ/SVZ, consistent with a role for this molecule in the intracortical guidance of cortical interneurons (Borrell & Marín, 2006; Stumm *et al.*, 2007). In mice deficient in this chemokine, or its receptor CXCR4, interneurons alter their tangential migratory routes and invade the CP prematurely (Stumm *et al.*, 2003; Tiveron *et al.*, 2006; Li *et al.*, 2008; Lopez-Bendito *et al.*, 2008).

But what are the factors that determine the choice of stream by migrating interneurons as they enter the cortex? We reasoned that there exist molecular/genetic differences between interneurons that underlie their choice of one of the three tangential pathways. Here, we sought to identify genes involved in migratory stream specification by comparing the gene expression profiles of cells in the PPL with those of cells migrating through the IZ in early corticogenesis. We performed laser capture microdissection (LCM) on the cortices of glutamic acid decarboxylase-67 (GAD-67)–green fluorescent protein (GFP) transgenic mice (Tamamaki *et al.*, 2003) in order to isolate interneuron-enriched populations of cells from the two zones. Subsequent microarray analysis revealed a large number of genes that are differentially expressed in the two cell populations, including those encoding cell surface proteins and regulators of intracellular signalling pathways. We focused on genes that are specifically upregulated in each stream, and examined their expression profiles by *in situ* hybridization. Our results support a role for a number of mostly novel genes in the migration of interneurons along specific routes.

## Materials and methods

### Animals

All experimental procedures were performed in accordance with the UK Animals (Scientific Procedures) Act 1986 and institutional guidelines. GAD67–GFP (Δneo) mice (Tamamaki *et al.*, 2003) were maintained on a C57/BL6J background. The day on which the vaginal plug was found was considered to be E0.5. Mice of both sexes were used in all experiments.

### LCM

Embryonic brains (E13.5) were dissected in RNase-free phosphate-buffered saline (PBS), placed in cryostat moulds, and frozen in Tissue-Tek OCT (Sakura Finetek Europe, Zoeterwoude, The Netherlands). Forebrains were sectioned, allowed to adhere to LCM membrane-mounted slides (Zeiss MicroImaging, Jena, Germany), and stored at −80 °C until use. For LCM, slides were individually thawed for 30 s, fixed in cold methanol for 1 min, and rinsed rapidly in PBS. Slides were dehydrated through 70–100% ethanol, and allowed to dry thoroughly (30 s–1 min). GAD67–GFP-positive cells were excised from the PPL and IZ within 15 min of drying, by use of a Zeiss Palm Microbeam system (Zeiss MicroImaging). GAD67–GFP-positive cells were allowed to adhere to capture tube lids (Zeiss MicroImaging). Tubes were placed on dry ice and kept at −80 °C until RNA extraction.

### RNA extraction and microarray analysis

Total RNA from cortical PPL-derived and IZ-derived cells was extracted immediately after collection with the Qiagen RNeasy Miniplus kit (Qiagen, Chatsworth, CA, USA). RNA was sent to the Wolfson Institute for Biomedical Research (UCL Genomics, London, UK) for cDNA production, hybridization, and scanning. The quality of the RNA was assessed with an Agilent bioanalyser nanochip (Agilent, Palo Alto, CA, USA). All RNA had 18S and 28S rRNA bands. RNA (100 ng per chip) was converted to single-strand, sense-strand cDNA with the Affymetrix Sense target labelling protocol and the Mouse Gene 1.0ST Array kit (Affymetrix, High Wycombe, UK). After fragmenting and end-labelling, the cDNA was hybridized to Mouse Gene Gene-1_0-st-v1 Arrays (Affymetrix) at 45 °C for 16 h according to the manufacturer’s instructions. The arrays were then washed and stained on the Fluidics station 450 with the hybridization, wash and stain kits, and scanned on the GeneChip Scanner 3000. Analysis of microarray data was performed at the Bloomsbury Centre for Bioinformatics, Department of Computer Science (UCL). Raw data were summarized and normalized with the rma algorithm (Irizarry *et al.*, 2003) implemented in the Affymetrix Expression Console software. limma (Linear Models for Microarray Analysis) (Smyth, 2004) was used to identify differentially expressed genes. limma applies a modified *t*-test to each probe set that uses an empirical Bayes approach for estimating sample variances. The moderated *t*-statistic calculated by limma is more robust than the ordinary *t*-statistic with small sample sizes. The *P*-values were corrected for multiple testing with the Benjamini–Hochberg correction, and a corrected *P*-value threshold of 0.01, together with a fold cut-off of > 2, was used to select differentially expressed genes.

### Fluorescence-activated cell sorting (FACS)

Timed pregnant dams were killed at E13.5 and E15.5. Embryonic brains were dissected in cold artificial cerebrospinal fluid. The forebrain was isolated, and the meninges, olfactory bulb and septum were removed. The cortex and ganglionic eminence (GE) were separated, and the (presumptive) hippocampus was separated from the cortex. Cortex and GE cells were dissociated by incubation in 0.05% trypsin with 100 μg/mL DNase I in Neurobasal medium (Invitrogen, Paisley, UK) at 37 °C for 15 min. Trypsinization was quenched by addition of neurobasal medium containing 10% heat-inactivated fetal bovine serum (Invitrogen) at 37 °C for 5 min. Cells were washed three times in Neurobasal medium (without fetal bovine serum) to remove serum for FACS. Cells were resuspended in Neurobasal medium without phenol red (Invitrogen) containing l-glutamine (Invitrogen) and B-27 supplement (1: 50; Invitrogen). Dissociated cells from 8 to 10 embryos were pooled for each FACS. FACS was performed by the Wolfson Scientific Support Services (UCL) with a MoFlo Sorter (Dako, Copenhagen, Denmark). A non-green embryo was used as a control for fluorescence. Cells were excited with a 488-nm argon laser and detected with a 530/40 (FL1) bandpass filter. A cell purity of 95–98.5% was obtained for each sorting.

### Quantitative polymerase chain reaction (qPCR) validation

For validation of the differentially expressed genes, qPCR was performed on 20 genes. Embryonic dissection, LCM and RNA extraction were performed as previously described, and RNA was treated with DNase I (Amplification grade; Invitrogen) to remove any remaining trace amounts of DNA. cDNA was generated with 20 ng of RNA by use of the Qiagen Whole Transcriptome Amplification Kit (Qiagen), as described in the manufacturer’s protocol. Primers for qPCR were designed by SigmaGenosys (Sigma-Aldrich, Poole, UK), and were as shown in Supporting Information Table S1. The qPCR reaction was performed with SYBR Green reagent (Sigma, Poole, UK) on a Chromo4 PTC-200 Real-Time PCR Detector system (Bio-Rad, Hercules, CA, USA). Polymerase chain reaction (PCR) conditions were 94 °C for 2 min, followed by 40 three-step cycles of 94 °C for 15 s, 60 °C for 30 s, and 72 °C for 30 s. Glyceraldehyde-3-phosphate dehydrogenase (GAPDH) and β-actin were used for endogenous reference gene controls. Each primer set amplified a single PCR product of predicted size as determined by melt-curve analysis following PCR and by agarose gel electrophoresis, and had approximately equal amplification efficiencies when validated with a serial dilution of representative cDNA. Each qPCR was performed in triplicate, and relative quantification was determined according to the ΔΔc(t) method (Livak & Schmittgen, 2001).

### In situ *hybridization*

*In situ* hybridization was performed as described previously (Faux *et al.*, 2010). Briefly, embryonic brains were dissected in PBS and fixed in 4% paraformaldehyde in PBS for 4 h at 4 °C, and this was followed by cryoprotection in 30% diethyl pyrocarbonate-treated sucrose in PBS overnight at 4 °C. Brains were frozen in Tissue-Tek OCT (Sakura Finetek) and sectioned with a cryostat (20 μm; Bright Instruments, Huntingdon, UK). Sections were dried at room temperature for 2 h before overnight incubation at 65 °C in hybridization buffer [1 × diethyl pyrocarbonate-treated ‘salts’ (200 mm NaCl, 5 mm EDTA, 10 mm Tris, pH 7.5, 5 mm NaH_2_PO_4_.2H_2_O, 5 mm Na_2_HPO_4_); 50% deionized formamide (Ambion, Austin, TX, USA); 0.1 mg/mL RNase-free yeast tRNA (Invitrogen); 1 × Denhardts (RNase/DNase-free; Invitrogen); 10% dextran sulphate (Sigma)] containing 100–500 ng/mL digoxigenin-labelled RNA probes. Probes were generated by linearization of plasmids with appropriate enzymes and reverse transcription PCR to obtain antisense probes. A number of probes were obtained from the Max-Planck Institute of Biophysical Chemistry, Göttingen, Germany (Supporting Information Table S2). Other probes (Lhx6 and Reelin) were a kind gift from N. Kessaris (Wolfson Institute, UCL, UK). Following hybridization, sections were washed three times in wash solution (50% formamide, 1 × SSC, 0.1% Tween-20) at 65 °C and twice at room temperature in 1 × MABT (20 mm maleic acid, 30 mm NaCl, 0.1% Tween-20) before being incubated in blocking solution [2% blocking reagent (Roche Applied Science, Burgess Hill, UK), 10% normal goat serum (Vector, Burlingame, CA, USA) in MABT] and then overnight in alkaline phosphatase-conjugated anti-digoxigenin antibody (1: 1500; Roche Applied Science). Nitro Blue tetrazolium chloride/5-bromo-4-chloro-3-indolyl phosphate diluted 1: 1000 in MABT with 5% poly(vinyl alcohol) was used for colorimetric detection at 37 °C for 8–20 h. Fast Red (Roche Applied Science) was used for fluorescent colour detection of probes by incubation in 100 mm Tris (pH 8.0) and 400 mm NaCl containing Fast Red for approximately 2 h at 37 °C. Fluorescent *in situ* hybridization was followed by immunohistochemical detection of GFP as described below. Sections were mounted with Glycergel Mounting Medium (Dako). Photographs were taken with a Leica DM microscope and a Leica DC 500 digital camera. All images were processed with Photoshop CS2 software (Adobe, San Jose, CA, USA).

### Immunohistochemistry

Embryonic brains and cryosections were prepared as previously described. Sections and dissociated cortices were blocked for 1 h in PBS containing 5% normal goat serum, and then incubated in rabbit polyclonal anti-Cnr1 (1: 100; Sigma-Aldrich) and rabbit polyclonal anti-Dab1 (1: 100; Sigma-Aldrich) at room temperature overnight. They were then washed in PBS and incubated in biotinylated goat anti-rabbit (1: 200; Vector Laboratories) for 2 h. Antibody staining was enhanced with a tyramide signal amplification system (Perkin Elmer, Boston, MA, USA), according to the manufacturer’s instructions. Sections were washed and incubated with 4′,6-diamidino-2-phenylindole (1: 20 000; Sigma-Aldrich). Images were collected with an SP2 Leica confocal microscope. Sequential images were subsequently reconstructed with Metamorph imaging software (Universal Imaging Corporation, West Chester, PA, USA).

## Results

### Isolation of PPL and IZ cells by LCM

**Figure 1:**
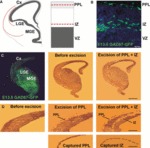
Tangential migration of interneurons into the cerebral cortex. (A) Schematic diagrams depicting the streams of migrating interneurons at E13.5. Red lines indicate the laminar positions of the PPL and IZ streams. (B) Coronal section through the cortex of an E13.5 GAD67–GFP transgenic mouse showing abundant migrating cells in the PPL and IZ streams. (C and D) Intact section through the forebrain of one hemisphere of an E13.5 GAD67–GFP transgenic mouse and after excision and capture of the PPL and IZ with a laser-capture microscope. Scale bars: (A) 100 μm; (C and D) 500 μm. Cx, cerebral cortex; LGE, lateral ganglionic eminence; VZ, ventricular zone.

Examination of the forebrains of GAD67–GFP transgenic mice during corticogenesis revealed cells undergoing tangential migration (Fig. 1A), as previously described (Tamamaki *et al.*, 2003; Métin *et al.*, 2006). At E13.5, GFP-positive (interneurons) cells were observed primarily in the PPL and IZ (Fig. 1B). Using LCM on coronally cut sections at this age, we isolated GAD67–GFP-enriched populations of cells from these two zones (Fig. 1C and D).

### Microarray analysis and validation

**Figure 2:**
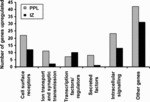
Numbers of genes upregulated in the PPL (grey bars) and IZ (black bars) migratory streams at E13.5. Genes were classified into the categories listed according to their molecular function.

In order to identify genes that may be involved in the choice of migratory stream by cortical interneurons, we compared gene expression in the PPL with that in cells isolated from the IZ by performing microarray analysis. Genes were considered to be differentially expressed if a greater than two-fold change in expression was found, together with a corrected *P*-value threshold of 0.05. The number of genes upregulated in PPL cells at E13.5 was 113, and that in IZ cells was 69. In order to examine more closely the overall changes in expression, genes were classified into six categories according to their molecular function (Fig. 2).

As an initial validation of our microarray data, we examined changes in expression of *Reelin* and *Dact1*, genes that are known to be strongly expressed in the PPL (Ogawa *et al.*, 1995; Faux *et al.*, 2010; respectively), *Robo2*, which is highly expressed in the IZ (Andrews *et al.*, 2007), and *Lhx6*, which is expressed by all cortical interneurons and has been shown to play a crucial role in their migration (Alifragis *et al.*, 2004). As expected, *Reelin* and *Dact1* were both expressed at higher levels in the PPL than in the IZ (Supporting Information Tables S6 and S7, respectively), whereas the opposite was the case for *Robo2* expression (Supporting Information Table S9). No significant changes were observed in the levels of expression of *Lhx6* or in specific interneuron subtype markers such as calbindin, calretinin, and somatostatin (data not shown).

Genes with higher expression levels in the PPL are listed in Supporting Information Tables S3–S8, and those with higher expression levels in the IZ are shown in Supporting Information Tables S9–S14. qPCR, carried out on a set of eight genes that showed higher expression levels in the PPL and on four that showed higher expression levels in the IZ at E13.5, was subsequently used to further validate the observed changes in expression (Tables 1 and 2). In this analysis, all genes were found to have fold changes in the same direction as the microarray. Interestingly, the fold changes observed by qPCR were much higher than those found with microarray, in agreement with previous observations (Faux *et al.*, 2010). Thus, our microarray analysis, together with the qPCR, identified a number of genes with upregulated expression in specific streams of migrating cortical interneurons.

**Table 1:** qPCR comparing expression profiles of cell surface receptor genes that are upregulated in PPL cells as compared with IZ cells

	Upregulated in PPL		Upregulated in IZ	
		
Gene	Microarray	qPCR	Microarray	qPCR

*Cdh8*			++	+++
*Cnr1*	+	+++		
***Dact1***	+	++		
*Epha3*			+	+++
*Flrt2*	+	+++		
***Lhx6***	NC	NC	NC	NC
*Mc4r*	+	+++		
*Nelf*	+	+++		
*Neuritin*			+	+++
*Ptpro*	+	+++		
***Reelin***	+	+++		
***Robo2***			+	+++

NC, no change; +, 2–5-fold greater; ++, 5–15-fold greater; +++, > 15-fold greater. Reference genes are in bold.

**Table 2:** qPCR comparing expression profiles of intracellular signalling genes that are upregulated in PPL cells as compared with IZ cells

	Upregulated in PPL		Upregulated in IZ	
		
Gene	Microarray	qPCR	Microarray	qPCR

*Cdc42ep3*			+	+++
*Dab1*	+	+++		
***Dact1***	+	++		
***Lhx6***	NC	NC	NC	NC
*Plcb1*			+	+++
*Rasgef1b*			+	+++
***Reelin***	+	+++		
***Robo2***			+	+++

NC, no change; +, 2–5-fold greater; ++, 5–15-fold greater; +++, > 15-fold greater. Reference genes are in bold.

### Cell surface receptors – new candidate genes for choice of migratory stream

Cell surface molecules are involved in essential developmental processes, such as migration, neurite outgrowth, and synapse formation. We present genes thought to be involved in cellular interactions that are enriched within the PPL (Supporting Information Table S3) and IZ (Supporting Information Table S9) interneuron streams at E13.5. With the exception of *Cnr1* (Morozov *et al.*, 2009), *EphA4* (Rudolph *et al.*, 2010), *Nrp1* (Marín *et al.*, 2001), *Robo1* (Andrews *et al.*, 2006), *Robo2* (Andrews *et al.*, 2008), and *Sstr2* (Beneyto *et al.*, 2011), these receptor genes have not previously been implicated in cortical interneuron development. However, two recent microarray studies that examined the differential expression of genes between interneurons and non-interneurons (presumptive pyramidal cells) in the cortex, and between cortical interneurons and GE cells, identified a number of cell surface receptor genes that are expressed in the cortical interneuron population (Batista-Brito *et al.*, 2008; Faux *et al.*, 2010). Some of these genes, including *Alcam*, *Cdh8*, *Cdh10*, *Csmd3*, *Fat3*, *Flrt2*, *Islr2*, *Mdga2*, *Nrn1*, *Nelf*, *Pcdh11x*, *Plxnc1*, *Ptpro*, and *Ptprz1*, were also identified here, and appear to be differentially expressed in the two migratory streams analysed (Supporting Information Tables S3 and S9). We subsequently performed *in situ* hybridization at E13.5 and E15.5, the early phase and mid-phase of tangential interneuron migration, for a selection of these genes to further confirm their expression in the PPL/MZ or IZ/SVZ.

**Figure 3:**
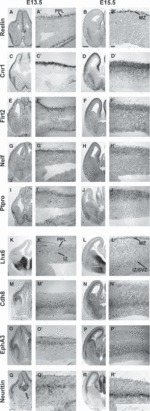
Expression of receptor genes in the interneuron migratory streams in the developing forebrain as seen by *in situ* hybridization at E13.5 and E15.5. A higher-magnification image of the cortex is shown beside each low-magnification panel of the forebrain. The upper panels show the expression of receptor genes in the PPL at E13.5 and MZ at E15.5. The lower panels show the expression of receptor genes predominantly in the IZ at E13.5 and E15.5. (A–B′) The expression of *Reelin* was used as an internal control, as it is known that it is expressed exclusively in cells (presumptive CR cells) in the PPL at E13.5 and in the MZ at E15.5. (C–J′) Expression of receptor genes *Cnr1* (C–D′), *Flrt2* (E–F′), *Nelf* (G–H′) and *Ptpro* (I–J) was localized predominantly within the PPL at E13.5 and within the MZ at E15.5. (K–L′) The interneuron marker *Lhx6* was also used as an internal control, as it is known to be expressed in both the PPL and IZ at E13.5 (K–K′), and more widely, but predominantly in the MZ and IZ/SVZ, at E15.5 (L–L′). (M–R′) Expression of the receptor genes *Cdh8* (M–N′), *EphA3* (O–P′) and *Neuritin* (Q–R′) within the IZ at E13.5 and within the IZ/SVZ at E15.5. Scale bar in A–B′: 200 μm.

Several receptor genes demonstrated strong specific expression in the PPL, but not in the IZ, at E13.5 (Fig. 3, upper panels). These included the G-protein-coupled cannabinoid 1 receptor gene (*Cnr1*) (Fig. 3C and C′), *Flrt2*, which encodes a glycosylated membrane protein that acts as a regulator of fibroblast growth factor signalling (Fig. 3E and E′), the nasal embryonic luteinizing hormone-releasing hormone factor gene (*Nelf*) (Fig. 3G and G′), and the protein tyrosine phosphotase receptor type O gene (*Ptpro*) (Fig. 3I and I′. At E15.5, all four genes showed increased expression in the MZ and CP, but not in the IZ/SVZ (Fig. 3D, D′, F, F′, H, H′, J, and J′), confirming previous cortical expression studies on *Cnr1* (Berrendero *et al.*, 1998) and *Nelf* (Kramer & Wray, 2001). We also observed weak expression of *Flrt2*, *Nelf* and *Ptpro* in the mantle zone of the MGE (Fig. 3E, F, G, H, I, and J), suggesting that they may have other developmental function(s) in addition to a specific role in tangential interneuron migration within the cortical PPL/MZ.

Specific expression of several receptor genes was evident in the IZ at E13.5 and E15.5 (Fig. 3, lower panels). These included the calcium-dependent cell adhesion glycoprotein cadherin-8 gene (*Cdh8*) (Fig. 3M–N′) (Takeichi, 1988), the ephrin-A tyrosine kinase receptor A3 gene (*EphA3*) (Fig. 3O–P′), and the glycosylphosphatidylinositol-linked neuritin receptor gene [(*Neuritin*, also known as *Cpg15*) (Fig. 3Q–R′)]. At E13.5, all three genes appeared to be specifically expressed in the IZ interneuron stream. At E15.5, they were expressed in the IZ/SVZ interneuron stream, but *Cdh8* and *EphA3* showed expanded expression within the IZ, confirming previous studies that localized the products of these genes in thalamocortical and commissural fibres (Korematsu & Redies, 1997; Kudo *et al.*, 2005). Furthermore, we observed varying levels of expression for *Cdh8* and *EphA3* in different sites of the subpallium (Fig. 3M, N, O, and P), in agreement with earlier reports (Korematsu & Redies, 1997; Kudo *et al.*, 2005).

### Cell signalling pathways – new candidate genes for choice of migratory stream

In addition to cell surface receptor genes that can directly affect cell migration and axon guidance events, our microarray analysis also identified various genes that modulate cell signalling pathways, which in turn can influence cell migration. In Supporting Information Tables S7 and S13, we list genes involved in intracellular signalling pathways whose expression is enriched within the PPL (Supporting Information Table S7) and IZ (Supporting Information Table S13). With the exception of *Dab1* (Hammond *et al.*, 2006), *Dact1* (Faux *et al.*, 2010), *Prkra* (Deng *et al.*, 2009), and *Syngap1* (Muhia *et al.*, 2010), all other genes have not previously been implicated in cortical interneuron development. However, recent microarray studies identified a number of signalling genes that are expressed in cortical interneuron populations (Batista-Brito *et al.*, 2008; Faux *et al.*, 2010). These included *Bub1*, *Cdc42ep3*, *Dab1*, *Dcn*, *Elmod1*, *Gng5*, *Plk2*, *Rasgef1b* and *Unc5d*, which were confirmed here and appear to be expressed differentially in the two streams (Supporting Information Tables S7 and S13).

**Figure 4:**
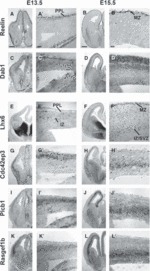
Expression of cell signalling genes in the interneuron migratory streams in the developing forebrain as seen by *in situ* hybridization at E13.5 and E15.5. A higher-magnification image of the cortex is shown beside each low-magnification panel of the forebrain. The upper panels show the expression of receptor genes in the PPL at E13.5 and in the MZ at E15.5. The lower panels show the expression of receptor genes predominantly in the IZ at E13.5 and E15.5. (A–B′) The expression of *Reelin* was used as an internal control, as it is known that it is expressed exclusively in cells (presumptive CR cells) in the PPL at E13.5 and in the MZ at E15.5. (C–D′) Expression of *Dab1* is observed only within the PPL at E13.5 and in the MZ and SP (after the splitting of the CP) at E15.5. (E–F′) The interneuron marker *Lhx6* was also used as an internal control, as it is known to be expressed in both the PPL and IZ at E13.5 (E–E′), and more widely, but predominantly in the MZ and IZ/SVZ, at E15.5 (F–F′). (G–L′) Expression of the cell signalling genes *Cdc42ep3* (G–H′), *Plcb1* (I–J′) and *Rasgef1b* (K–L′) was observed predominantly within the IZ at E13.5, and within the IZ/SVZ at E15.5. Scale bar in A–B′: 200 μm.

Using *in situ* hybridization, we examined the expression of several signalling genes that showed significant levels of expression in either the PPL/MZ (Fig. 4, upper panels) or the IZ (Fig. 4, lower panels). For example, *Dab1* showed specific expression in the PPL, but not in the IZ, at E13.5 (Fig. 4C and C′) and, at E15.5, it showed stronger expression in the MZ and CP, as well as in the SP interneuron stream (Fig. 4D and D′). Three signalling genes that showed specific expression in the IZ interneuron stream at E13.5 included the CDC42 effector protein (Rho GTPase binding) 3 gene (*Cdc42ep3*) (Fig. 4G and G′), the phosphoinositide-specific phospholipase Cβ1 gene (*Plcb1*) (Fig. 4I and I′), and the gene encoding RasGEF1b, a guanine-nucleotide exchange factor (*Rasgef1b*) (Fig. 4K and K′). At E15.5, both *Cdc42ep3* and *Rasgef1b* expression appeared to be limited to the IZ/SVZ interneuron stream (Fig. 4H, H, respectively), whereas *Plcb1* showed an expanded expression band around the IZ (Fig. 4J and J′), in agreement with previous findings (Watanabe *et al.*, 1998).

**Figure 5:**
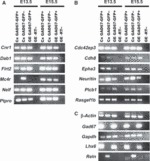
Agarose gel electophoresis of qPCR products. qPCR was performed on cortex-derived and GE-derived GAD67–GFP-positive and GAD67–GFP-negative cells at E13.5 and E15.5. The genes examined are listed next to the gel bands. (A) Genes shown to be upregulated in the cortical PPL/MZ. Nearly all genes were expressed in the three samples at both ages. The only exception was *Mc4r*, which was found to be expressed only in GAD67–GFP-negative cells (presumptive pyramidal neuron progenitors) in the cortex at E13.5. (B) Genes shown to be upregulated in the IZ. (C) Control genes. Cx, cortex; -RT, without reverse transcriptase (negative control).

Although PPL and IZ cell populations isolated by LCM contain predominantly interneurons, they are likely to also contain other cell types. Thus, the PPL population undoubtedly also included CR cells, and the IZ samples were likely to also contain pyramidal neurons migrating through this zone *en route* to the CP. To confirm whether all genes identified in each of the two migratory streams are indeed expressed in interneurons, we first assessed their expression by PCR in FACS populations of GAD67–GFP-positive and GFP-negative cells derived from the cortex and GAD67–GFP-positive GE cells at E13.5 and E15.5 (Fig. 5). With the single exception of *Mc4r* at E13.5, all other genes were shown to be expressed in both cortical and GE GAD67–GFP-positive populations at both ages.

**Figure 6:**
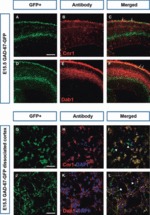
Expression of Cnr1 and Dab1 in interneurons. (A–F) Coronal sections from GAD67–GFP-positive mice at E15.5 were immunostained with anti-Cnr1 (B) and anti-Dab1 (E) (red). Co-localization (yellow; arrows) of Cnr1 and Dab1 with GFP is evident in some neurons in the MZ in the merged panels (C and F). (G–L) Dissociated cortical cell cultures prepared from E15.5 GAD67–GFP-positive cortices were immunostained with anti-Cnr1 (H) and anti-Dab1 (K). GFP single-positive cells are indicated by arrowheads, and Cnr1/GFP or Dab1/GFP double-positive cells are indicated by arrows in the merged panels (I and L). Scale bars: (A) 200 μm; (G) 15 μm.

To further confirm the expression of some of these genes in interneurons within specific tangential migratory streams, we performed double-labelling experiments. For example, we carried out immunofluorescence investigations for Cnr1 and Dab1 (red) on sections taken from GAD67–GFP brains at E15.5 (Fig. 6A–F). Co-localization between GFP and either Cnr1 or Dab1 (yellow) was observed in the MZ, but not in the IZ/SVZ (Fig. 6C and F), suggesting that some interneurons in the MZ express Cnr1 and Dab1. This was confirmed further by Cnr1 and Dab1 immunohistochemistry on GAD67–GFP-positive dissociated cortical cell cultures at E15.5 (Fig. 6G–L).

Taken together, our findings have identified a number of genes whose expression is upregulated in specific interneuron populations, either within the PPL/MZ or within the IZ/SVZ, during corticogenesis. These genes may be potentially important for the migration of these cells and their choice of migratory stream.

## Discussion

Since the discovery in the late 1990s that cortical interneurons have their origins in the subpallium, at least in rodents, numerous studies have traced in detail their long and tortuous migratory paths (reviewed in Marín & Rubenstein, 2003; Métin *et al.*, 2006; Hernández-Miranda *et al.*, 2010). These cells move round the corticostriatal notch, enter the neocortex, and migrate initially along well-defined tangential streams in the PPL/MZ, IZ, and SP, before moving radially to populate the CP. To date, only a few signalling and guidance molecules have been found that directly control their migration, and almost nothing is known about their choice of migratory stream. In the present study, we utilized a whole genome analysis of mRNAs expressed in the PPL and IZ at an early stage of corticogenesis. We focused our analysis on groups of genes (cell surface receptors and intracellular signalling) that are known to play a role in neuronal migration, both in the cortex and in other areas of the developing brain, as well as on genes that are thought to be involved in migration-related events such as neurite outgrowth and guidance, cell adhesion, and intracellular signalling. This analysis identified a number of genes that are differentially upregulated in the two streams and may be involved in the choice of pathway by migrating cortical interneurons.

### Cell surface receptors

Many of the cell surface genes found to be upregulated in cortical interneurons have previously been shown to play a role in cell migration, either directly or indirectly, by regulating specific events that are essential for this process, such as neurite outgrowth and branching. One of the cell surface receptor genes identified here, *Cnr1*, appeared to be specifically upregulated in the PPL/MZ. Cnr1 is one of the most widely expressed G-protein-coupled receptors in the mammalian brain (Herkenham *et al.*, 1991), and has been shown previously to be expressed in interneurons (Katona *et al.*, 1999). In addition, it has been identified in pyramidal cells (Berrendero *et al.*, 1998), in a subclass of hippocampal GABAergic interneurons containing the neuropeptide cholecystokinin (Katona *et al.*, 1999), and in neuronal elements of the striatum (Rodriguez *et al.*, 2001), a finding confirmed in our present study. Schizophrenia subjects show a decrease in Cnr1 mRNA and protein levels in the prefrontal cortex (Eggan *et al.*, 2008), as well as diminished parvalbumin mRNA in the same region (Hashimoto *et al.*, 2003). In an attempt to investigate the role of Cnr1 signalling, a recent analysis of *Cnr1* null mice showed decreased parvalbumin immunoreactivity in the cortex and striatum (Fitzgerald *et al.*, 2011), highlighting its importance in interneuron development. Recent evidence has also pointed to a role for *Cnr1* in neuronal migration, as loss of function in a neural stem cell line and rostral migratory stream decreased migration and, conversely, direct activation resulted in increased migration (Oudin *et al.*, 2011).

Our analysis also identified cell surface molecules, such as Nrp1, Robo1 and Robo2, that have previously been shown to be expressed in cortical interneurons and play an important role in their development (Marín *et al.*, 2001; Andrews *et al.*, 2006, 2008). Furthermore, it revealed the expression of genes encoding cell surface proteins, including *Cdh8*, *Nelf*, *Pcdh19*, *Plxnd1*, *Sema5a*, *Sorl1*, and *Vcan*, which have not previously been shown to be expressed in interneurons, but are known to play key roles in cell migration and neuronal differentiation in the developing brain. Thus, *Cdh8* is expressed in the IZ/SVZ, but not specifically in interneurons within this zone (Korematsu & Redies, 1997), and recent studies have suggested that it regulates mossy fiber fasciculation and targeting (Bekirov *et al.*, 2008). *Nelf* plays a role in the outgrowth of olfactory axons and migration of gonadotropin-releasing hormone neurons (Kramer & Wray, 2000; Xu *et al.*, 2010), whereas *Neuritin* functions to coordinately regulate the growth of dendritic and axonal arbours and to promote synaptic maturation (Nedivi *et al.*, 1998; Cantallops *et al.*, 2000; Javaherian & Cline, 2005). *Plxnd1* has been shown to control migration of thymocytes (Choi *et al.*, 2008), and *Sema5a* acts as an axon guidance cue for axial motor neurons (Hilario *et al.*, 2009); like *Vcan*, it has been reported to induce neuronal differentiation and promote neurite outgrowth (Wu *et al.*, 2004). Whether these genes have similar functions in interneuron migration and development requires further experimental assessment.

### Intracellular signalling pathways

In addition to cell surface receptor genes that can directly affect cell migration and axon guidance events, our microarray analysis also identified various genes that modulate cell signalling pathways, which, in turn, can influence developmental events such as migration, neurite outgrowth, and synaptic function. Included in this group of genes is *Dab1*, which encodes an intracellular adaptor that is expressed in cells that respond to Reelin (Howell *et al.*, 1997). As such, it is an essential component of the Reelin signalling pathway, and is required for correct radial migration of cortical pyramidal neurons (Howell *et al.*, 1997; Franco *et al.*, 2011). It has been reported (Rice *et al.*, 1998; Trommsdorff *et al.*, 1999), and confirmed in our present *in situ* hybridization experiments, that *Dab1* is also expressed in the MGE, where interneurons commence their migration towards the cortex. The importance of Reelin function in cortical interneuron development has been demonstrated in organotypic slice culture and *in vivo* experiments (Morante-Oria *et al.*, 2003). Subsequent studies of *Dab1*^*−/−*^ mice showed abnormal cortical interneuron layering (Pla *et al.*, 2006). Furthermore, pulse labelling experiments indicated that early-born (E12.5) interneurons in *Dab1* mutant brains show layer inversion, whereas the lamination of late-born interneurons is unaffected (Hammond *et al.*, 2006), suggesting that expression of *Dab1* in early-born interneurons is required for correct cortical layering.

In the present study, we have also identified intracellular signalling molecules known to play a role in developmental events, including neuronal migration, that have not previously been shown to be expressed in interneurons. A number of the genes encoding these molecules, including *Gpr12*, *Plcb1*, and *Syngap1*, have been implicated in neuronal process development (Tanaka *et al.*, 2007; Spires *et al.*, 2005; Carlisle *et al.*, 2008), and *Rgs6* has been implicated in neuronal differentiation (Liu *et al.*, 2002). Like *Sorbs2* and *Prkce*, they have been shown to have a key role in cell migration (Roignot *et al.*, 2010; Solecki *et al.*, 2004). Whether these genes fulfil such roles in cortical interneuron development remains to be determined.

Our PCR analysis of FACS populations of GAD67–GFP-positive and GAD67–GFP-negative cells derived from the cortex indicated that all but one of the genes found to be upregulated in the PPL and IZ are expressed in interneurons, but the majority are also expressed in non-interneurons. It is possible that the expression of genes identified in cellular elements surrounding migrating interneurons contributes to a permissive environment for their migration. For example, Robo receptors are expressed on axons coursing through the IZ and in interneurons (Andrews *et al.*, 2006, 2008), and Robo homophilic–heterophilic interactions have been shown to be important for neurite outgrowth (Hivert *et al.*, 2002). Thus, Robo receptors may be required for interneuron migration along IZ axons, similar to what has been postulated for the neural adhesion molecule TAG-1 (Denaxa *et al.*, 2001). Similarly, Reelin, which is predominantly expressed by CR cells in the PPL/MZ and is known to act as a chemoattractant for migrating neurons (Ogawa *et al.*, 1995), may also contribute to a permissive environment that promotes the migration of interneurons. In support of this possibility is our finding that interneurons express the Reelin signalling molecule Dab1.

Interestingly, although a significant proportion of the genes found to be upregulated in the PPL and IZ migratory streams have so far not been associated with interneuron development, several have been linked to human neurological disorders. Thus, mutations in *Nelf* have been identified in patients with normosmic hypogonadotropic hypogonadism and Kallmann syndrome (Xu *et al.*, 2010, 2011). Similarly, *Nrp1* (Supporting Information Table S9), which shows a similar expression pattern to *Nelf* within gonadotropin-releasing hormone neurons, but is also expressed by cortical interneurons (Marín *et al.*, 2001), has been suggested to play a role in the aetiology of hypogonadotropic hypogonadism (Cariboni *et al.*, 2011). Mutations in *Cdh8* and *Plcb1* are associated with susceptibility to autism and the development of early-onset epilepsy, respectively (Pagnamenta *et al.*, 2011; Kurian *et al.*, 2010). Furthermore, alterations in mRNA levels of Cnr1, CDC42 and neuritin have been linked to the development of schizophrenia (Eggan *et al.*, 2008; Hill *et al.*, 2006; Chandler *et al.*, 2010), and alterations in the level of Sorl1 mRNA have been linked to the development of Alzheimer’s disease (Kölsch *et al.*, 2009). The observations that many of the genes identified here are associated with neurological disorders suggests that disruption of cortical interneuron development may contribute to the underlying aetiology of these disorders. Thus, future studies should aim at elucidating the function of these genes in cortical interneuron migration and development.

## Abbreviations

CP: cortical plate
CR: Cajal–Retzius
E: embryonic day
FACS: fluorescence-activated cell sorting
GAD-67: glutamic acid decarboxylase-67
GAPDH: glyceraldehyde-3-phosphate dehydrogenase
GE: ganglionic eminence
GFP: green fluorescent protein
IZ: intermediate zone
LCM: laser capture microdissection
MGE: medial ganglionic eminence
MZ: marginal zone
PBS: phosphate-buffered saline
PCR: polymerase chain reaction
PPL: preplate
qPCR: quantitative polymerase chain reaction
SP: subplate
SVZ: subventricular zone

## Supporting information

**Table S1.** qPCR primer details.

**Table S2.** Genepaint PCR *in situ* probe details.

**Table S3.** Cell surface receptors with upregulated expression in the cortical PPL.

**Table S4.** Ion transport and synaptic transmission genes with upregulated expression in the cortical PPL.

**Table S5.** Transcription factors/regulators with upregulated expression in the cortical PPL.

**Table S6.** Secreted factors with upregulated expression in the cortical PPL.

**Table S7.** Intracellular signalling molecules with upregulated expression in the cortical PPL.

**Table S8.** Other genes from different classes with upregulated expression in the cortical PPL.

**Table S9.** Cell surface receptors with upregulated expression in the cortical IZ.

**Table S10.** Ion transport and synaptic transmission genes with upregulated expression in the cortical IZ.

**Table S11.** Transcription factors/regulators with upregulated expression in the cortical IZ.

**Table S12.** Secreted factors with with upregulated expression in the cortical IZ.

**Table S13.** Intracellular signalling molecules with upregulated expression in the cortical IZ.

**Table S14.** Other genes from different classes with upregulated expression in the cortical IZ.

As a service to our authors and readers, this journal provides supporting information supplied by the authors. Such materials are peer-reviewed and may be re-organized for online delivery, but are not copy-edited or typeset by Wiley-Blackwell. Technical support issues arising from supporting information (other than missing files) should be addressed to the authors.
