# Supplementary material for: Differential gene expression in migratory streams of cortical interneurons
Source: Eur J Neurosci. 2011 Nov;34(10):1584–94. doi: 10.1111/j.1460-9568.2011.07896.x (PMC3401901; doi:10.1111/j.1460-9568.2011.07896.x)
Supplement: Supplementary file 1 [file ejn0034-1584-SD1.doc]

SUPPORTING INFORMATION TABLE S1. qPCR primer details. Sequence (5’-3’), amplicon length, and accession number of primers

| Primer | Sequence | Amplicon | Accession number |
| --- | --- | --- | --- |
| Cdc42ep3-F | CCAAGACCCCAATTTACCTGAAA | 136 | NM_026514 |
| Cdc42ep3-R | CCCTCTTTGCCGATGTGTATAGT |
| Cdh8-F | TGCATGAGGCAGATAATGACCC | 129 | NM_007667 |
| Cdh8-R | TCTGGTCTGAGTCTGATGTGG |
| Cnr1-F | AAGTCGATCTTAGACGGCCTT | 123 | NM_007726 |
| Cnr1-R | TCCTAATTTGGATGCCATGTCTC |
| Dab1-F | CCTCACTTCATCCCACAGAAAAA | 100 | NM_177259 |
| Dab1-R | CTGCCATAGGTATCTGCCATTG |
| EphA3-F | TTCTCCATCTCCGGTGAAAACA | 103 | NM_010140 |
| EphA3-R | ACCTCCCGACCAGAACATAGG |  |
| Flrt2-F | ATGGGCCTACAGACTACAAAGT | 125 | NM_201518 |
| Flrt2-R | CAGCGGCATACACTAGGGC |
| Gad67-F | CACAGGTCACCCTCGATTTTT | 176 | NM_008077 |
| Gad-67-R | ACCATCCAACGATCTCTCTCATC |
| Gapdh-F | AGGGCATCTTGGGCTACAC | 123 | NM_008084 |
| Gapdh-R | CATACCAGGAAATGACGTTGA |
| Lhx6-F | GCCGCATCCATTACGACACC | 112 | AJ000337 |
| Lhx6-R | TGGCTGGCTTGGGCTGAC |
| Mc4r-F | CCCGGACGGAGGATGCTAT | 101 | NM_016977 |
| Mc4r-R | TCGCCACGATCACTAGAATGT |
| Nelf-F | AGCCATATCGAACTTCCGCAA | 134 | NM_020276 |
| Nelf-R | AAGTGTCGGCTTTCATAGGGG |
| Nrn1-F | GCGGTGCAAATAGCTTACCTG | 151 | NM_153529 |
| Nrn1-R | CGGTCTTGATGTTCGTCTTGTC |
| Plcb1-F | TCAGTCTTGTCGGAAGGTTGC | 80 | NM_019677 |
| Plcb1-R | CACTTGAGAGCTTGAGGGTTG |
| Ptpro-F | ACAGACGCTTTCATTTCCCCG | 243 | AF135166 |
| Ptpro-R | TGGGCTTCTCCGTCCTATCG |
| Rasgef1b-F | ACCGAAACCTCTACCAGTCC | 102 | NM_145839 |
| Rasgef1b-R | ACCGAAACCTCTACCAGTCC |
| Reln-F | TGCGAGTGGGTGAGGTGTAT | 153 | NM_011261 |
| Reln-R | AGCTATGCTTGACCGTTGCTC |

F, Forward; R, Reverse

SUPPORTING INFORMATION TABLE S2. Genepaint PCR insitu probe details

|  |  |  |  |  |  |  |
| --- | --- | --- | --- | --- | --- | --- |
|  |  |  |  |  |  |  |
| Gene Paint Set ID | Accession | Gene code | Gene name | Length (bps) | Anti-sense | Sense |
| EH1012 | NM_026514 | Cdc42ep3 | Mus musculus CDC42 effector protein (Rho GTPase binding) 3 | 1273 | T3 | T7 |
| MH1196 | NM_007667 | Cdh8 | cadherin 8 | 1019 | T7 | Sp6 |
| MH99 | Y18374 | Cnr1 | cannabinoid receptor 1 | 1533 | T3 | T7 |
| MH356 | NM_010014 | Dab1 | disabled homolog 1 (Drosophila) | 689 | Sp6 | T7 |
| MH2232 | NM_010140 | Epha3 | Eph receptor A3 | 842 | Sp6 | T7 |
| EH1969 | NM_201518 | Flrt2 | fibronectin leucine rich transmembrane protein 2 | 2927 | T7 | Sp6 |
| MH409 | AF201662 | Mc4r | melanocortin 4 receptor | 1087 | Sp6 | T7 |
| EH1979 | NM_020276 | Nelf | nasal embryonic LHRH factor | 3000 | T7 | Sp6 |
| EH1679 | NM_153529 | Nrn1 | Neuritin | 2000 | T7 | Sp6 |
| MH1626 | NM_019677 | Plcb1 | phospholipase C beta 1 | 933 | Sp6 | T7 |
| EH765 | NM_011216 | Ptpro | Mus musculus protein tyrosine phosphatase, receptor type, O | 750 | T3 | T7 |
| EH724 | NM_145839 | Rasgef1b | Mus musculus RasGEF domain family, member 1B | 1400 | T7 | Sp6 |
|  |  |  |  |  |  |  |
|  |  |  |  |  |  |  |

SUPPORTING INFORMATION TABLE S3. Cell surface receptors with upregulated expression in cortical PPL

| Gene | Fold Change | p-value | Gene name | Accession  Number (NCBI) | Role in migration/  Neurological disorders |
| --- | --- | --- | --- | --- | --- |
| Alcam | 2.14 | 0.0019 | activated leukocyte cell adhesion molecule | NM_009655 | Heffron and Golden, 2000.  (Faux et al., 2010) |
| Cd34 | 2.38 | 0.0005 | CD34 antigen | NM_133654 |  |
| Cdh13 | 2.73 | 0.0004 | cadherin 13 | NM_019707 |  |
| **Cnr1** | 2.08 | 0.0014 | cannabinoid receptor 1 | NM_007726 | Neurite outgrowth (Zorina et al., 2010) |
| Csmd3 | 2.36 | 0.0004 | CUB and Sushi multiple domains 3 | NM_001081391 | Links with autism (Floris et al., 2008); Epliepsy (Shimizu et al., 2003).  (Faux et al., 2010) |
| Eltd1 | 2.17 | 0.0079 | EGF, latrophilin seven transmembrane domain containing 1 | NM_133222 |  |
| Fat3 | 3.58 | 0.0002 | FAT tumor suppressor homolog 3 | NM_001080814 | Neurite interactions? (Nagae et al., 2007).  (Faux et al., 2010) |
| Fat4 | 2.50 | 0.0003 | FAT tumor suppressor homolog 4 | NM_183221 |  |
| **Flrt2** | 3.34 | 0.0001 | fibronectin leucine rich transmembrane protein 2 | NM_201518 | Neural crest cell migration (Gong et al., 2009). (Faux et al., 2010) |
| Hnt | 2.05 | 0.0152 | neurotrimin | NM_172290 |  |
| Ifngr2 | 2.31 | 0.0002 | interferon gamma receptor 2 | NM_008338 |  |
| Islr2 | 3.53 | 0.0016 | immunoglobulin superfamily containing leucine-rich repeat 2 | NM_177193 | Axonal projection, guidance defects in null (mandai et al., 2009).  (Faux et al., 2010) |
| Itm2a | 5.65 | 0.0026 | integral membrane protein 2A | NM_008409 |  |
| Lrfn5 | 2.39 | 0.0012 | leucine rich repeat and fibronectin type III domain containing 5 | NM_178714 |  |
| Mc4r | 2.68 | 0.0009 | melanocortin 4 receptor | NM_016977 |  |
| Mdga2 | 4.56 | 0.0117 | MAM domain containing glycosyl-phosphatidylinositol anchor 2 | NM_207010 | Suggested role in neuronal migration  (Litwack et al., 2004).  (Faux et al., 2010) |
| **Nelf** | 2.16 | 0.0172 | nasal embryonic LHRH factor | NM_001039386 | GnRH neuronal migration  (Xu et al., 2009).  (Faux et al., 2010) |
| Pcdh11x | 3.39 | 0.031 | protocadherin 11 X-linked | NM_001081385 | associated with susceptibility to late-onset Alzheimer's disease (Carrasquillo et al., 2009).  (Faux et al., 2010) |
| Pcdh19 | 2.01 | 0.0022 | protocadherin 19 | NM_001105245 | Linked with Epilepsy and mental retardation (Hynes et al., 2010) |
| Plxnc1 | 2.41 | 0.0029 | plexin C1 | NM_018797 | Uesugi et al., 2009.  (Faux et al., 2010) |
| Plxnd1 | 2.85 | 0.0069 | plexin D1 | NM_026376 | Choi et al., 2008 |
| **Ptpro** | 2.51 | 0.0021 | protein tyrosine phosphatase, receptor type, O | NM_011216 | Neurite outgrowth (Gonzalez-Brito and Bixby, 2009).  (Faux et al., 2010) |
| Ptprz1 | 2.39 | 0.0010 | protein tyrosine phosphatase, receptor type Z, polypeptide 1 | NM_001081306 | Role in neuron migration (Maeda and Noda, 1998). (Faux et al., 2010) |
| Robo1 | 2.17 | 0.01 | roundabout homolog 1 (Drosophila) | NM_019413 | Andrews et al., 2006, 2008.  (Faux et al., 2010) |
| Vcan | 2.13 | 0.0037 | versican | NM_001081249 | Promotes neurite outgrowth (Wu et al., 2004) |

Genes in bold were confirmed by in situ hybridisation

SUPPORTING INFORMATION TABLE S4. Ion transport and synaptic transmission genes with upregulated expression in cortical PPL

| Gene | Fold Change | p-value | Gene name | Accession  Number (NCBI) | Role in migration/  Neurological disorders |
| --- | --- | --- | --- | --- | --- |
| Gamma-aminobutyric acid receptors | | | | | |
| Gabrb2 | 5.82 | 0.0001 | gamma-aminobutyric acid (GABA-A) receptor, subunit beta 2 | NM_008070 |  |
| Glutamate receptors | | | | | |
| Gria3 | 2.81 | 0.0008 | glutamate receptor, ionotropic, AMPA3 (alpha 3) | NM_016886 | Links witith cognitive impairment (Wu et al., 2007) |
| Grik3 | 2.1 | 0.0035 | glutamate receptor, ionotropic, kainate 3 | NM_001081097 | Link with schizophrenia (Ahmad et al., 2009) |
| Grin2b | 2.08 | 0.0035 | glutamate receptor, ionotropic, NMDA2B (epsilon 2) | NM_008171 |  |
| Calcium channels | | | | | |
| Cacna1e | 2.46 | 0.0006 | calcium channel, voltage-dependent, R type, alpha 1E subunit | NM_009782 |  |
| Cacng4 | 2.46 | 0.0013 | calcium channel, voltage-dependent, gamma subunit 4 | NM_019431 |  |
| Patassium Channels | | | | | |
| Kcna4 | 2.05 | 0.01 | potassium voltage-gated channel, shaker-related | NM_021275 |  |
| Kcnt2 | 2.04 | 0.0055 | potassium channel, subfamily T, member 2 | NM_001081027 |  |
| Sodium channels | | | | | |
| Scn2a1 | 4.72 | 0.0004 | sodium channel, voltage-gated, type II, alpha 1 | NM_001099298 |  |
| Synapse function | | | | | |
| Lrrtm2 | 2.41 | 0.0015 | leucine rich repeat transmembrane neuronal 2 | NM_178005 | Synapse formation  (De Wit et al., 2009) |
| Nrxn1 | 2.39 | 0.0006 | neurexin I | NM_020252 | Synapse formation  (De Wit et al., 2009) |

SUPPORTING INFORMATION TABLE S5. Transcription factors/ regulators with upregulated expression in cortical PPL

| Gene | Fold Change | p-value | Gene name | Accession  Number (NCBI) | Role in migration/  Neurological disorders |
| --- | --- | --- | --- | --- | --- |
| Adamts3 | 6.45 | 0.0001 | ADAM metallopeptidase with thrombospondin type 1 motif, 3 | NM_001081401 |  |
| Adamts17 | 2.38 | 0.0129 | ADAM metallopeptidase with thrombospondin type 1 motif, 17 | NM_001033877 |  |
| Camta1 | 2.60 | 0.0171 | calmodulin binding transcription activator 1 | NM_001081557 |  |
| Ldb2 | 3.84 | 0.0001 | LIM domain binding 2 | NM_001077398 | Cell migration  (Storbeck et al., 2009) |
| Sox5 | 2.14 | 0.0042 | SRY-box containing gene 5 | NM_011444 | Migration and differentiation (Kwan et al., 2008) |
| Tceal5 | 2.16 | 0.0009 | transcription elongation factor A (SII)-like 5 | NM_177919 |  |
| Zeb1 | 2.05 | 0.0036 | zinc finger E-box binding homeobox 1 | NM_011546 |  |

SUPPORTING INFORMATION TABLE S6. Secreted factors with upregulated expression in cortical PPL

| Gene | Fold Change | p-value | Gene name | Accession  Number (NCBI) | Role in migration/  Neurological disorders |
| --- | --- | --- | --- | --- | --- |
| Fgf14 | 2.05 | 0.0264 | fibroblast growth factor 14 | NM_207667 | Role in synaptic transmission (Xiao et al., 2007) |
| Lgi1 | 2.1 | 0.027 | leucine-rich repeat LGI family, member 1 | NM_020278 | Link with Epilepsy and neurtie outgrowth (Owour et al., 2009) |
| Nrg3 | 3.01 | 0.0173 | neuregulin 3 | NM_008734 | Cell migration (Anton et al., 2004) |
| Npy | 2.25 | 0.0102 | neuropeptide Y | NM_023456 | Cell migration (Decressac et al., 2009) |
| Postn | 2.75 | 0.002 | periostin | NM_015784 | Cell migration (Li et al., 2010) |
| Reln | 3.58 | 0.0001 | Reelin | NM_011261 | Neuronal migration (Hevner et al., 2004; Cariboni et al., 2005) |
| Rspo3 | 4.17 | 0.0006 | R-spondin 3 | NM_028351 |  |
| Sepp1 | 2.30 | 0.0095 | selenoprotein P, plasma, 1 | NM_009155 | Cell migration (Copper et al., 2008) |

SUPPORTING INFORMATION TABLE S7. Intracellular signalling molecules with upregulated expression in cortical PPL

| Gene | Fold Change | p-value | Gene name | Accession  Number (NCBI) | Role in migration/  Neurological disorders |
| --- | --- | --- | --- | --- | --- |
| Arhgap20 | 2.19 | 0.0048 | Rho GTPase activating protein 20 | NM_175535 | Neurite outgrowth (Yamada et al., 2005).  (Faux et al., 2010) |
| Arhgap29 | 2.89 | 0.0022 | Rho GTPase activating protein 29 | NM_172525 |  |
| **Dab1** | 2.79 | 0.0016 | disabled homolog 1 | NM_177259 | Neuronal positioning (Olson et al., 2006).  (Faux et al., 2010) |
| Dact1 | 3.36 | 0.0023 | dapper homolog 1, antagonist of beta-catenin (xenopus) | NM_021532 | Role in dendritic arbourisation (Okerlund et al., 2010).  (Faux et al., 2010) |
| Dcn | 3.27 | 0.0001 | Decorin | NM_007833 | Role in axon growth (Minor et al., 2008).  (Faux et al., 2010) |
| Elmod1 | 2.66 | 0.0003 | ELMO domain containing 1 | NM_177769 | (Faux et al., 2010) |
| Gng11 | 2.05 | 0.0206 | guanine nucleotide binding protein (G protein), gamma 11 | NM_025331 |  |
| Gpr12 | 2.23 | 0.0011 | G-protein coupled receptor 12 | NM_001010941 | Role in neurite outgrowth (Tanaka et al., 2007) |
| Mapk3 | 2.60 | 0.0001 | mitogen activated protein kinase 3 | NM_011952 |  |
| Myo9a | 3.14 | 0.0146 | myosin IXa | XM_981204 | Gene found within the Bardet-Biedl syndrome (BBS4) region  (Gorman et al., 1999) |
| Plk2 | 2.93 | 0.0003 | polo-like kinase 2 | NM_152804 | (Batista-Brito et al., 2008) |
| Ppp2r2c | 2.28 | 0.0056 | protein phosphatase 2 (formerly 2A), regulatory subunit B (PR 52), gamma isoform | NM_172994 |  |
| Prkra | 2 | 0.0052 | protein kinase, interferon inducible double stranded RNA dependent activator | NM_011871 |  |
| Prkce | 2.41 | 0.0039 | protein kinase C, epsilon | NM_011104 | Role in cell migration (Solecki et al., 2004) |
| Prkcz2 | 3.34 | 0.0032 | protein kinase C, zeta 2 | XM_994291 |  |
| **Ptpro** | 2.51 | 0.0021 | protein tyrosine phosphatase, receptor type, O | NM_011216 | Neurite outgrowth (Gonzalez-Brito and Bixby, 2009).  (Faux et al., 2010) |
| Ptprz1 | 2.39 | 0.0010 | protein tyrosine phosphatase, receptor type Z, polypeptide 1 | NM_001081306 | Role in neuron migration (Maeda and Noda, 1998). (Faux et al., 2010) |
| Rgs5 | 2.17 | 0.0064 | regulator of G-protein signalling 5 | NM_009063 |  |
| Rgs6 | 2.04 | 0.0010 | regulator of G-protein signalling 6 | NM_015812 | Role in neuronal differentiation (Liu et al., 2002) |
| Rasgrp3 | 2.07 | 0.0065 | RAS, guanyl releasing protein 3 | NM_207246 |  |
| Stk32b | 2.57 | 0.003 | serine/threonine kinase 32B | NM_022416 |  |
| Syngap1 | 6.14 | 0.0033 | synaptic Ras GTPase activating protein 1 homolog (rat) | XM_985548 | Role in spine morphology (Carlisle et al., 2008) |

Genes in bold were confirmed by in situ hybridisation

SUPPORTING INFORMATION TABLE S8. Other genes from different classes with upregulated expression in cortical PPL

| Gene | Fold Change | p-value | Gene name | Accession  Number (NCBI) | Role in migration/  Neurological disorders |
| --- | --- | --- | --- | --- | --- |
| Actl6b | 2.01 | 0.0034 | actin-like 6B | NM_031404 |  |
| Ankrd12 | 2.06 | 0.0038 | ankyrin repeat domain 12 | NM_001025572 |  |
| Bgn | 2.02 | 0.0008 | biglycan | NM_007542 |  |
| Chst1 | 2.49 | 0.0107 | carbohydrate (keratan sulfate Gal-6) sulfotransferase 1 | NM_023850 |  |
| Cnih2 | 2.05 | 0.0028 | cornichon homolog 2 (Drosophila) | NM_009920 |  |
| Colec12 | 2.57 | 0.0031 | collectin sub-family member 12 | NM_130449 |  |
| Crabp1 | 2.12 | 0.0034 | cellular retinoic acid binding protein I | NM_013496 | Role in neuron differentiation (Uhrig et al., 2008) |
| Ccnyl1 | 2.05 | 0.0041 | cyclin Y-like 1 | NM_001097644 |  |
| Dnm3 | 2.38 | 0.0018 | dynamin 3 | NM_001038619 |  |
| Fabp3 | 2.08 | 0.0439 | fatty acid binding protein 3 | NM_010174 |  |
| Frmd3 | 3.68 | 0.0021 | FERM domain containing 3 | NM_172869 |  |
| Hmcn1 | 3.14 | 0.0114 | hemicentin 1 | NM_001024720 |  |
| Hspa1l | 3.48 | 0.0122 | heat shock protein 1-like | NM_013558 |  |
| Ifitm3 | 3.73 | 0.0064 | interferon induced transmembrane protein 3 | NM_025378 | Role in cell migration (Tanaka et al., 2005) |
| Igf2bp1 | 2.69 | 0.0001 | insulin-like growth factor 2 mRNA binding protein 1 | NM_008081 |  |
| Igfbp3 | 2.81 | 0.0050 | insulin-like growth factor binding protein 3 | NM_008343 |  |
| Ina | 2.62 | 0.0022 | internexin neuronal intermediate filament protein, alpha | NM_146100 | Role in cell migration (Ortino et al., 2003) |
| Lgals1 | 3.36 | 0.006 | lectin, galactose binding, soluble 1 | NM_008495 | Role in axonal regeneration (Horie et al., 2004) |
| Lxn | 2.45 | 0.0014 | latexin | NM_016753 |  |
| Lum | 2.22 | 0.015 | lumican | ENSMUST00000038160 | Role in neurite outgrowth (Cole and McCabe, 1991) |
| March4 | 2.35 | 0.0233 | membrane-associated ring finger (C3HC4) 4 | NM_001045533 |  |
| Mosc2 | 2.17 | 0.0072 | MOCO sulphurase C-terminal domain containing 2 | NM_133684 |  |
| Mpped1 | 2.14 | 0.0116 | metallophosphoesterase domain containing 1 | NM_172610 |  |
| Nin | 2.62 | 0.0005 | Ninein | NM_008697 | Role in neuronal differentiation (Ohama and Hayashi, 2009) |
| Nme5 | 3.23 | 0.0234 | expressed in non-metastatic cells 5 | NM_080637 |  |
| Parp8 | 2.26 | 0.0003 | poly (ADP-ribose) polymerase family, member 8 | NM_001081009 |  |
| Pfkp | 2.31 | 0.0034 | phosphofructokinase, platelet | NM_019703 |  |
| Prcp | 2.23 | 0.0165 | prolylcarboxypeptidase (angiotensinase C) | NM_028243 | Neuronal function (Ward et al., 2007) |
| Prkar2b | 2.23 | 0.0037 | protein kinase, cAMP dependent regulatory, type II beta | NM_011158 | Defective motor behaviour in null mice  (Brandon et al., 1998) |
| Prkcz2 | 3.34 | 0.0032 | protein kinase C, zeta 2 | XM_994291 | Role in cell motility (Della Peruta et al., 2010) |
| Rab12 | 2.04 | 0.0007 | RAB12, member RAS oncogene family | NM_024448 |  |
| S100a6 | 2.71 | 0.0145 | S100 calcium binding protein A6 (calcyclin) | NM_011313 | Cell migration (Yamashita et al., 1997) |
| Sh3gl2 | 3.05 | 0.0001 | SH3-domain GRB2-like 2 | NM_019535 |  |
| Slap1 | 3.73 | 0.0008 | src-like adaptor | NM_001029841 |  |
| Slc44a1 | 2.41 | 0.0185 | solute carrier family 44, member 1 | NM_133891 |  |
| Snord116 | 5.09 | 0.0115 | small nucleolar RNA, C/D box 116 | NR_002895 |  |
| Sparcl1 | 5.83 | 0.3680 | SPARC-like 1 (mast9, hevin) | NM_010097 | Role in migration (Gongidi et al., 2004) |
| Tanc2 | 2.71 | 0.0133 | tetratricopeptide repeat, ankyrin repeat and coiled-coil containing 2 | NM_181071 |  |
| Thra | 2.01 | 0.0018 | thyroid hormone receptor alpha | NM_178060 |  |
| Ube2v2 | 3.38 | 0.0012 | ubiquitin-conjugating enzyme E2 variant 2 | NM_023585 |  |
| Zfp294 | 2.19 | 0.0037 | zinc finger protein 294 | NM_001081068 | Role in neurodegeneration (Chu et al., 2009) |
| Zfp804a | 2.42 | 0.0078 | zinc finger protein 804A | NM_175513 |  |

SUPPORTING INFORMATION TABLE S9. Cell surface receptors with upregulated expression in cortical IZ

| Gene | Fold Change | p-value | Gene name | Accession  Number (NCBI) | Role in migration/  Neurological disorders |
| --- | --- | --- | --- | --- | --- |
| **Cdh8** | 6.2 | 0.0109 | Cadherin 8 | NM_001039154 | (Faux et al., 2010) |
| Cdh10 | 2-27 |  | Cadherin 10 | NM_009865 | (Faux et al., 2010) |
| **Epha3** | 2.79 | 0.0324 | Eph receptor A3 | NM_010140 | Axon guidance (Jayasena et al., 2005) |
| Epha4 | 2.48 | 0.0327 | Eph receptor A4 | NM_007936 | Interneuron migration (Rudolph et al, 2010).  (Faux et al., 2010) |
| Gpm6b | 2.31 | 0.0283 | glycoprotein m6b | NM_023122 |  |
| **Nrn1** | 2.1 | 0.0355 | neuritin 1 | NM_153529 | Neurite outgrowth (Fujino et al., 2008).  (Faux et al., 2010) |
| Nrp1 | 3.43 | 0.0133 | neuropilin 1 | NM_008737 | Interneuron migration (Marín et al., 2001).  (Faux et al., 2010) |
| Robo2 | 2.64 | 0.0249 | roundabout homolog 2 (Drosophila) | NM_175549 | Axon guidance (Plachez et al., 2008) |
| Sema5a | 2.67 | 0.0099 | sema domain, seven thrombospondin repeats (type 1 and type 1-like), transmembrane domain (TM) and short cytoplasmic domain | NM_009154 | Axon guidance  (Hilario et al., 2009) |
| Sema6d | 2.78 | 0.0083 | sema domain, transmembrane domain (TM), and cytoplasmic domain, (semaphorin) 6D | NM_172537 |  |
| Sorl1 | 2.1 | 0.0026 | sortilin-related receptor, LDLR class A repeats-containing | NM_011436 | Role in Alzheimer disease (Ma et al., 2009) |
| Sstr2 | 5.39 | 0.0067 | somatostatin receptor 2 | NM_001042606 | Altered expression in schizophrenia. (Beneyto et al., 2011) |

Genes in bold were confirmed by in situ hybridisation

SUPPORTING INFORMATION TABLE S10. Ion transport and synaptic transmission genes with upregulated expression in cortical IZ

| Gene | Fold Change | p-value | Gene name | Accession  Number (NCBI) | Role in migration/  Neurological disorders |
| --- | --- | --- | --- | --- | --- |
| Synapse function | | | | | |
| Gjd2 | 2.11 | 0.0005 | gap junction protein, delta 2 | NM_010290 |  |
| Slc17a6 | 4.5 | 0.0067 | solute carrier family 17 (sodium-dependent inorganic phosphate cotransporter), member 6 | NM_080853 |  |

SUPPORTING INFORMATION TABLE S11. Transcription factors/ regulators with upregulated expression in cortical IZ

| Gene | Fold Change | p-value | Gene name | Accession  Number (NCBI) | Role in migration/  Neurological disorders |
| --- | --- | --- | --- | --- | --- |
| Baz2b | 2.3 | 0.0122 | bromodomain adjacent to zinc finger domain, 2b | NM_001001182 |  |
| Eomes | 5.58 | 0.03 | eomesodermin homolog (Xenopus laevis) | NM_010136 | Role in neurogenesis (Arnold et al., 2008) |
| Neurod1 | 6.68 | 0.002 | neurogenic differentiation 1 | NM_010894 | Role in neuronal differentiation (Roybon et al., 2010) |
| Neurod4 | 2.11 | 0.0069 | neurogenic differentiation 4 | NM_007501 | Role in neuronal differentiation (Inoue et al., 2002) |
| Neurog2 | 2.64 | 0.0148 | neurogenin 2 | NM_009718 |  |
| Nhlh1 | 3.2 | 0.0048 | nescient helix loop helix 1 | NM_010916 | Neuronal migration (Schmid et al., 2007) |
| Nr2e1 | 2.19 | 0.023 | nuclear receptor subfamily 2, group E, member 1 | NM_152229 |  |
| Nr4a2 | 2.08 | 0.0007 | nuclear receptor subfamily 4, group A, member 2 | NM_013613 | Role in neuronal migration (Wallen et al., 1999) |
| Tcfap2d | 4.44 | 0.002 | transcription factor AP-2, delta | NM_153154 |  |
| Zfp369 | 2.23 | 0.0005 | zinc finger protein 369 | NM_178364 |  |

SUPPORTING INFORMATION TABLE S12. Secreted factors with upregulated expression in cortical IZ

| Gene | Fold Change | p-value | Gene name | Accession  Number (NCBI) | Role in migration/  Neurological disorders |
| --- | --- | --- | --- | --- | --- |
| Sema3C | 3.68 | 0.0047 | sema domain, immunoglobulin domain (Ig), short basic domain, secreted, (semaphorin) 3C | NM_013657 | Cell migration (Esselens et al., 2010) |

SUPPORTING INFORMATION TABLE S13. Intracellular signalling molecules with upregulated expression in cortical IZ

| Gene | Fold Change | p-value | Gene name | Accession  Number (NCBI) | Role in migration/  Neurological disorders |
| --- | --- | --- | --- | --- | --- |
| Bub1 | 2.28 | 0.0216 | budding uninhibited by benzimidazoles 1 homolog (S. cerevisiae) | NM_009772 | (Faux et al., 2010) |
| **Cdc42ep3** | 2.73 | 0.0056 | CDC42 effector protein (Rho GTPase binding) 3 | NM_026514 | Altered Cdc42signalling in Schizophrenia (Ide and , Lewis, 2010).  (Batista-Brito et al., 2008) |
| Cks1b | 2.07 | 0.0299 | CDC28 protein kinase 1b | NM_016904 |  |
| Crkrs | 2.12 | 0.0088 | Cdc2-related kinase, arginine/serine-rich | NM_026952 |  |
| Gng5 | 2.35 | 0.0111 | guanine nucleotide binding protein (G protein), gamma 5 subunit | NM_010318 | (Faux et al., 2010) |
| **Plcb1** | 2.62 | 0.0089 | phospholipase C, beta 1 | NM_019677 | Dendrite spine morphology (Spires et al., 2005) |
| Rab8b | 2.39 | 0.0098 | RAB8B, member RAS oncogene family | NM_173413 |  |
| **Rasgef1b** | 2.17 | 0.0085 | RasGEF domain family, member 1B | NM_145839 | (Faux et al., 2010) |
| Ttk | 2.19 | 0.0458 | Ttk protein kinase | NM_009445 |  |
| Sh3bgrl2 | 2.25 | 0.026 | SH3 domain binding glutamic acid-rich protein like 2 | NM_172507 |  |
| Socs6 | 2.83 | 0.0108 | suppressor of cytokine signalling 6 | NM_018821 |  |
| Sorbs2 | 2.25 | 0.006 | sorbin and SH3 domain containing 2 | NM_172752 | Role in cell migration (Roignot et al., 2010) |
| Unc5d | 7.78 | 0.001 | unc-5 homolog D (C. elegans) | NM_153135 | Role in neuronal survival (Tang et al., 2008) .  (Faux et al., 2010) |

Genes in bold were confirmed by in situ hybridisation

SUPPORTING INFORMATION TABLE S14. Other genes from different classes with upregulated expression in cortical IZ

| Gene | Fold Change | p-value | Gene name | Accession  Number (NCBI) | Role in migration/  Neurological disorders |
| --- | --- | --- | --- | --- | --- |
| Abcd2 | 12.38 | 0.0016 | ATP-binding cassette, sub-family D (ALD), member 2 |  |  |
| Casc5 | 2.3 | 0.0357 | cancer susceptibility candidate 5 | NM_029617 |  |
| Cdc26 | 2.03 | 0.0046 | cell division cycle 26 | NM_139291 |  |
| Cenpk | 2.16 | 0.0302 | NM_021790 | NM_021790 |  |
| Cep350 | 3.48 | 0.001 | centrosomal protein 350 | XM_129509 |  |
| Chd7 | 2.57 | 0.0002 | chromodomain helicase DNA binding protein 7 | NM_001081417 | Defects in neural stem cell proliferation (Layman et al., 2009) |
| Dbf4 | 2.01 | 0.0285 | DBF4 homolog (S. cerevisiae) | NM_013726 |  |
| Eif5 | 2.97 | 0.0015 | eukaryotic translation initiation factor 5 | NM_173363 |  |
| Fbxo5 | 2.23 | 0.0329 | F-box protein 5 | NM_025995 |  |
| Gas2l3 | 2.38 | 0.0041 | growth arrest-specific 2 | NM_001033331 |  |
| Ipo7 | 4.47 | 0.0171 | importin 7 | NM_181517 |  |
| Kif4 | 2.01 | 0.0216 | kinesin family member 4 | NM_008446 |  |
| Kif11 | 3.49 | 0.0182 | kinesin family member 11 | NM_010615 | Role in axon, and dendritic function (Yoon et al., 2005) |
| Lyrm5 | 2.39 | 0.0227 | LYR motif containing 5 | NM_133688 |  |
| Msh2 | 2.19 | 0.0269 | mutS homolog 2 (E. coli) | NM_008628 |  |
| Nsfl1c | 2.06 | 0.0391 | NSFL1 (p97) cofactor (p47) | NM_198326 |  |
| Nup43 | 2.35 | 0.0148 | nucleoporin 43 | NM_145706 |  |
| Ofd1 | 1.93 | 0.0009 | oral-facial-digital syndrome 1 gene homolog (human) | NM_177429 |  |
| Paip1 | 2.03 | 0.0301 | polyadenylate binding protein-interacting protein 1 | NM_145457 |  |
| Pam | 2.38 | 0.0009 | peptidylglycine alpha-amidating monooxygenase | NM_013626 |  |
| Plekhf2 | 2.31 | 0.0003 | pleckstrin homology domain containing, family F (with FYVE domain) member 2 | NM_175175 |  |
| Prr11 | 2.93 | 0.0153 | proline rich 11 | NM_175563 |  |
| Rlbp1 | 1.93 | 0.0008 | retinaldehyde binding protein 1-like 1 | NM_020599 |  |
| Rnasel | 2.81 | 0.0012 | ribonuclease L (2', 5'-oligoisoadenylate synthetase-dependent) | NM_011882 |  |
| Slc30a10 | 2.38 | 0.0053 | solute carrier family 30, member 10 | NM_001033286 |  |
| Tagln3 | 2.06 | 0.0237 | transgelin 3 | NM_019754 |  |
| Tcp1 | 2.81 | 0.0284 | t-complex protein 1 | NM_013686 |  |
| Timm23 | 2.06 | 0.0232 | translocase of inner mitochondrial membrane 23 homolog (yeast) | NM_016897 |  |
| Tmem77 | 2.028 | 0.0039 | transmembrane protein 77 | NM_026013 |  |
| Twf1 | 2.16 | 0.0223 | twinfilin, actin-binding protein, homolog 1 (Drosophila | NM_008971 |  |
| Ust | 2.11 | 0.0051 | uronyl-2-sulfotransferase | NM_177387 | Ishii & Maeda, 2008 |
| Uxt | 3.46 | 0.0179 | ubiquitously expressed transcript | NM_013840 |  |

**Supporting Information References**

Ahmad, Y., Bhatia, M.S., Mediratta, P.K., Sharma, K.K., Negi, H., Chosdol, K. & Sinha, S. (2009) Association between the ionotropic glutamate receptor kainate3 (GRIK3) Ser310Ala polymorphism and schizophrenia in the Indian population. *World J Biol Psychiatry*., **10**, 330-333.

Andrews, W., Liapi, A., Plachez, C., Camurri, L., Zhang, J., Mori, S., Murakami, F., Parnavelas, J.G., Sundaresan, V. & Richards L.J. (2006) Robo1 regulates the development of major axon tracts and interneuron migration in the forebrain. *Development,* **133**, 2243-2252.

Andrews, W., Barber, M., Hernadez-Miranda, L.R., Xian, J., Rakic, S., Sundaresan, V., Rabbitts, T.H., Pannell, R., Rabbitts, P., Thompson, H., Erskine, L., Murakami, F. & Parnavelas, J.G. (2008) The role of Slit-Robo signalling in the generation, migration and morphological differentiation of cortical interneurons. *Dev Biol*, **313**, 648-658.

Anton, E.S., Ghashghaei, H.T., Weber, J.L., McCann, C., Fischer, T.M., Cheung, I.D., Gassmann, M., Messing, A., Klein, R., Schwab, M.H., Lloyd, K.C. & Lai, C. (2004) Receptor tyrosine kinase ErbB4 modulates neuroblast migration and placement in the adult forebrain. *Nat Neurosci*, **7**, 1319-1328.

Arnold, S.J., Huang, G.J., Cheung, A.F., Era, T., Nishikawa, S., Bikoff, E.K., Molnár, Z., Robertson, E.J. & Groszer, M. (2008) The T-box transcription factor Eomes/Tbr2 regulates neurogenesis in the cortical subventricular zone. *Genes Dev*, **22**, 2479-2484.

Batista-Brito, R., Machold, R., Klein, C. & Fishell, G. (2008) Gene expression in cortical interneuron precursors is prescient of their mature function. *Cereb. Cortex*, **18**, 2306-2317.

Beneyto, M., Morrism H,M,, Rovenskym K,C, & Lewis DA. (2011) Lamina- and cell-specific alterations in cortical somatostatin receptor 2 mRNA expression in schizophrenia. Neuropharmacology, **12.029**

Brandon, E.P., Logue, S.F., Adams, M.R., Qi, M., Sullivan, S.P., Matsumoto, A.M., Dorsa, D.M., Wehner, J.M., McKnight, G.S. & Idzerda, R.L. (1998) Defective motor behavior and neural gene expression in RIIbeta-protein kinase A mutant mice. *J Neurosci*., **18**, 3639-3649.

Cariboni, A., Rakic, S., Liapi, A., Maggi, R., Goffinet, A. & Parnavelas, J.G. (2005) Reelin provides an inhibitory signal in the migration of gonadotropin-releasing hormone neurons. *Development,* **132**, 4709-4718.

Carlisle, H.J., Manzerra, P., Marcora, E. & Kennedy, M.B. (2008) SynGAP regulates steady-state and activity-dependent phosphorylation of cofilin. *J. Neurosci*., **28**, 13673-13683.

Carrasquillo, M.M., Zou, F., Pankratz, V.S., Wilcox, S.L., Ma, L., Walker, L.P., Younkin, S.G., Younkin, C.S., Younkin, L.H., Bisceglio, G.D., Ertekin-Taner, N., Crook, J.E., Dickson, D.W., Petersen, R.C., Graff-Radford, N.R. & Younkin, S.G. (2009) Genetic variation in PCDH11X is associated with susceptibility to late-onset Alzheimer's disease. *Nat. Genet*., **41**, 192-198.

Choi, Y.I., Duke-Cohan, J.S., Ahmed, W.B., Handley, M.A., Mann, F., Epstein, J.A., Clayton, L.K. & Reinherz, E.L. (2008) PlexinD1 glycoprotein controls migration of positively selected thymocytes into the medulla. *Immunity*, **29**, 888-898.

Chu, J., Hong, N.A., Masuda, C.A., Jenkins, B.V., Nelms, K.A., Goodnow, C.C., Glynne, R.J., Wu, H., Masliah, E., Joazeiro, C.A. & Kay SA. (2009) A mouse forward genetics screen identifies LISTERIN as an E3 ubiquitin ligase involved in neurodegeneration. *Proc. Natl. Acad. Sci. U. S. A*., **106**, 2097-2103.

Cole, G.J. & McCabe, C.F. (1991) Identification of a developmentally regulated keratan sulfate proteoglycan that inhibits cell adhesion and neurite outgrowth. *Neuron*, **7**, 1007-1018.

Cooper, M.L., Adami, H.O., Grönberg, H., Wiklund, F., Green, F.R. & Rayman, M.P. (2008) Interaction between single nucleotide polymorphisms in selenoprotein P and mitochondrial superoxide dismutase determines prostate cancer risk. *Cancer Res*., **68**, 10171-10177.

Decressac, M., Prestoz, L., Veran, J., Cantereau, A., Jaber, M. & Gaillard, A. (2009) Neuropeptide Y stimulates proliferation, migration and differentiation of neural precursors from the subventricular zone in adult mice. *Neurobiol. Dis*., **34**, 441-449.

Della, M., Giagulli, C., Laudanna, C., Scarpa, A. & Sorio, C. (2010) RHOA and PRKCZ control different aspects of cell motility in pancreatic cancer metastatic clones. *Mol. Cancer*. **9**, 61.

de Wit, J., Sylwestrak, E., O'Sullivan, M.L., Otto, S., Tiglio, K., Savas, J.N., Yates, J.R. 3rd, Comoletti, D., Taylor, P. & Ghosh, A. (2009) LRRTM2 interacts with Neurexin1 and regulates excitatory synapse formation. *Neuron*., **64**, 799-806.

Esselens, C., Malapeira, J., Colomé, N., Casal, C., Rodríguez-Manzaneque, J.C., Canals, F. & Arribas, J. (2010) The cleavage of semaphorin 3C induced by ADAMTS1 promotes cell migration. *J. Biol. Chem*., **285**, 2463-2473.

Faux, C., Rakic, S., Andrews, W., Yanagawa, Y., Obata, K. & Parnavelas, J.G. (2010) Differential gene expression in migrating cortical interneurons during mouse forebrain development. *J. Comp. Neurol.,* **518**, 1232-1248.

Floris, C., Rassu, S., Boccone, L., Gasperini, D., Cao, A. & Crisponi, L. (2008) Two patients with balanced translocations and autistic disorder: CSMD3 as a candidate gene for autism found in their common 8q23 breakpoint area. *Eur. J. Hum. Genet*., **16**, 696-704.

Fujino, T., Wu, Z., Lin, W.C., Phillips, M.A. & Nedivi, E. (2008) cpg15 and cpg15-2 constitute a family of activity-regulated ligands expressed differentially in the nervous system to promote neurite growth and neuronal survival. *J. Comp. Neurol*., **507**, 1831-1845.

Gong, S.G., Mai, S., Chung, K. & Wei, K. (2009) Flrt2 and Flrt3 have overlapping and non-overlapping expression during craniofacial development. *Gene Expr. Patterns*, **9**, 497-502.

Gongidi, V., Ring, C., Moody, M., Brekken, R., Sage, E.H., Rakic, P. & Anton, E.S. (2004) SPARC-like 1 regulates the terminal phase of radial glia-guided migration in the cerebral cortex. *Neuron*, **41**, 57-69.

Gonzalez-Brito, M.R. & Bixby, J.L. (2009) Protein tyrosine phosphatase receptor type O regulates development and function of the sensory nervous system. *Mol. Cell. Neurosci*., **42**, 458-465.

Gorman, S.W., Haider, N.B., Grieshammer, U., Swiderski, R.E., Kim, E., Welch, J.W., Searby, C., Leng, S., Carmi, R., Sheffield, V.C. & Duhl, D.M. (1999) The cloning and developmental expression of unconventional myosin IXA (MYO9A) a gene in the Bardet-Biedl syndrome (BBS4) region at chromosome 15q22-q23. *Genomics*, **59**, 150-160.

[Heffron, D.S](http://www.ncbi.nlm.nih.gov/pubmed?term="Heffron DS"%5BAuthor%5D). & Golden, J.A. (2000) DM-GRASP is necessary for nonradial cell migration during chick diencephalic development. [*J. Neurosci*.](javascript:AL_get(this, 'jour', 'J Neurosci.');), **20**, 2287-2294.

Heng, J.I., Nguyen, L., Castro, D.S., Zimmer, C., Wildner, H., Armant, O., Skowronska-Krawczyk, D., Bedogni, F., Matter, J.M., Hevner, R. & Guillemot, F. (2008) Neurogenin 2 controls cortical neuron migration through regulation of Rnd2. *Nature*, **455**, 114-118.

Hevner, R.F., Daza, R.A., Englund, C., Kohtz, J. & Fink, A. (2004) Postnatal shifts of interneuron position in the neocortex of normal and reeler mice: evidence for inward radial migration. *Neuroscience*, **124**, 605-618.

Hilario, J.D., Rodino-Klapac, L.R., Wang, C. & Beattie, C.E. (2009) Semaphorin 5A is a bifunctional axon guidance cue for axial motoneurons in vivo. *Dev. Biol*., **326**, 190-200.

Horie, H., Kadoya, T., Hikawa, N., Sango, K., Inoue, H., Takeshita, K., Asawa, R., Hiroi, T., Sato, M., Yoshioka, T., & Ishikawa, Y. (2004) Oxidized galectin-1 stimulates macrophages to promote axonal regeneration in peripheral nerves after axotomy. *J. Neurosci*. **24**, 1873-1880.

Hynes, K., Tarpey, P., Dibbens, L.M., Bayly, M.A., Berkovic, S.F., Smith, R., Raisi, Z,A,, Turner, S.J., Brown, N.J., Desai, T.D., Haan, E., Turner, G., Christodoulou, J., Leonard, H., Gill, D., Stratton, M.R., Gecz, J. & Scheffer, I.E. (2009) Epilepsy and mental retardation limited to females with PCDH19 mutations can present de novo or in single generation families. *J. Med. Genet*. **47**, 211-216.

Ide, M. & Lewis, D.A. (2010) Altered Cortical CDC42 Signalling Pathways in Schizophrenia: Implications for Dendritic Spine Deficits. *Biol. Psychiatry*, **68**, 25-32.

Inoue, T., Hojo, M., Bessho, Y., Tano, Y., Lee, J.E. & Kageyama, R. (2002) Math3 and NeuroD regulate amacrine cell fate specification in the retina. *Development*, **129**, 831-842.

Ishii, M. & Maeda, N. (2008) Oversulfated chondroitin sulfate plays critical roles in the neuronal migration in the cerebral cortex. *J. Biol. Chem*., **283**, 32610-32620.

Jayasena, C.S., Flood, W.D. & Koblar, S.A. (2005) High EphA3 expressing ophthalmic trigeminal sensory axons are sensitive to ephrin-A5-Fc: implications for lobe specific axon guidance. Neuroscience, 135, 97-109.

Kwan, K.Y., Lam, M.M., Krsnik, Z., Kawasawa, Y.I., Lefebvre, V. & Sestan, N. (2008) SOX5 postmitotically regulates migration, postmigratory differentiation, and projections of subplate and deep-layer neocortical neurons. *Proc. Natl. Acad. Sci. U. S. A*., **105**, 16021-16026.

Layman, W.S., McEwen, D.P., Beyer, L.A., Lalani, S.R., Fernbach, S.D., Oh, E.., Swaroop, A., Hegg, C.C., Raphael, Y., Martens, J.R. & Martin, D.M. (2009) Defects in neural stem cell proliferation and olfaction in Chd7 deficient mice indicate a mechanism for hyposmia in human CHARGE syndrome. *Hum. Mol. Genet*. **18**, 1909-1923.

Li, G., Jin, R., Norris, R.A., Zhang, L., Yu, S., Wu, F., Markwald, R.R., Nanda, A., Conway, S.J., Smyth, S.S. & Granger, D.N. (2010) Periostin mediates vascular smooth muscle cell migration through the integrins alphavbeta3 and alphavbeta5 and focal adhesion kinase (FAK) pathway. *Atherosclerosis*, **208**, 358-365.

Litwack, E.D., Babey, R., Buser, R., Gesemann, M. & O'Leary, D.D. (2004) Identification and characterization of two novel brain-derived immunoglobulin superfamily members with a unique structural organization. *Mol. Cell. Neurosci.* **25**, 263-274.

Liu, Z., Chatterjee, T.K. & Fisher, R.A. (2002) RGS6 interacts with SCG10 and promotes neuronal differentiation. Role of the G gamma subunit-like (GGL) domain of RGS6. *J. Biol. Chem*. **277**, 37832-37839.

Ma, Q.L., Galasko, D.R., Ringman, J.M., Vinters, H.V., Edland, S.D., Pomakian, J., Ubeda, O.J., Rosario, E.R., Teter, B., Frautschy, S.A. & Cole, G.M. (2009) Reduction of SorLA/LR11, a sorting protein limiting beta-amyloid production, in Alzheimer disease cerebrospinal fluid. *Arch. Neurol*., **66**, 448-457.

Maeda, N. & Noda, M. (1998) Involvement of receptor-like protein tyrosine phosphatase zeta/RPTPbeta and its ligand pleiotrophin/heparin-binding growth-associated molecule (HB-GAM) in neuronal migration. *J. Cell. Biol*., **142**, 203-216.

Mandai, K., Guo, T., St Hillaire, C., Meabon, J.S., Kanning, K.C., Bothwell, M. & Ginty, D.D. (2009) LIG family receptor tyrosine kinase-associated proteins modulate growth factor signals during neural development. *Neuron*, **63**, 614-627.

Marín, O., Yaron, A., Bagri, A., Tessier-Lavigne, M. & Rubenstein, J.L. (2001) Sorting of striatal and cortical interneurons regulated by semaphorin-neuropilin interactions. *Science*, **293**, 872-875.

Minor, K., Tang, X., Kahrilas, G., Archibald, S.J., Davies, J.E. & Davies, S.J. (2008) Decorin promotes robust axon growth on inhibitory CSPGs and myelin via a direct effect on neurons. *Neurobiol, Dis*., **32**, 88-95.

Nagae, S., Tanoue, T. & Takeichi, M. (2007) Temporal and spatial expression profiles of the Fat3 protein, a giant cadherin molecule, during mouse development. *Dev. Dyn*. **236**, 534-543.

Ohama, Y. & Hayashi, K. (2009) Relocalization of a microtubule-anchoring protein, ninein, from the centrosome to dendrites during differentiation of mouse neurons. *Histochem. Cell. Biol*., **132**, 515-524.

Okerlund, N.D., Kivimäe, S., Tong, C.K., Peng, I.F., Ullian, E.M. & Cheyette, B.N. (2010) Dact1 is a postsynaptic protein required for dendrite, spine, and excitatory synapse development in the mouse forebrain. *J. Neurosci*., **30**, 4362-4368.

Olson, E.C., Kim, S. & Walsh, C.A. (2006) Impaired neuronal positioning and dendritogenesis in the neocortex after cell-autonomous Dab1 suppression. *J. Neurosci*., **26**, 1767-1775.

Ortino, B., Inverardi, F., Morante-Oria, J., Fairén, A. & Frassoni, C. (2003) Substrates and routes of migration of early generated neurons in the developing rat thalamus. *Eur. J. Neurosci*., **18**, 323-332.

Owuor, K., Harel, N.Y., Englot, D.J., Hisama, F., Blumenfeld, H. & Strittmatter, S.M. (2009) LGI1-associated epilepsy through altered ADAM23-dependent neuronal morphology. *Mol. Cell. Neurosci*., **42**, 448-457.

Plachez, C., Andrews, W., Liapi, A., Knoell, B., Drescher, U., Mankoo, B., Zhe, L., Mambetisaeva, E., Annan, A., Bannister, L., Parnavelas, J.G., Richards, L.J. & Sundaresan, V. (2008) Robos are required for the correct targeting of retinal ganglion cell axons in the visual pathway of the brain. *Mol. Cell. Neurosci*., **37**, 719-730.

Roignot, J., Taïeb, D., Suliman, M., Dusetti, N.J., Iovanna, J.L. & Soubeyran, P. (2010) CIP4 is a new ArgBP2 interacting protein that modulates the ArgBP2 mediated control of WAVE1 phosphorylation and cancer cell migration. *Cancer Lett*., **288**, 116-123.

Roybon, L., Mastracci, T.L., Ribeiro, D., Sussel, L., Brundin, P. & Li, J.Y. (2010) GABAergic differentiation induced by Mash1 is compromised by the bHLH proteins Neurogenin2, NeuroD1, and NeuroD2. *Cereb. Cortex*. **20**, 1234-1244.

Rudolph, J., Zimmer, G., Steinecke, A., Barchmann, S. & Bolz, J. (2010) Ephrins guide migrating cortical interneurons in the basal telencephalon. *Cell. Adh. Migr*., **4**, 400-408.

Schmid, T., Krüger, M. & Braun, T. (2007) NSCL-1 and -2 control the formation of precerebellar nuclei by orchestrating the migration of neuronal precursor cells. *J. Neurochem*., **102**, 2061-2072.

Shimizu, A., Asakawa, S., Sasaki, T., Yamazaki, S., Yamagata, H., Kudoh, J., Minoshima, S., Kondo, I. & Shimizu, N. (2003) A novel giant gene CSMD3 encoding a protein with CUB and sushi multiple domains: a candidate gene for benign adult familial myoclonic epilepsy on human chromosome 8q23.3-q24.1. *Biochem. Biophys. Res. Commun*., **309**, 143-154.

Solecki, D.J., Mode,l L., Gaetz, J., Kapoor, T.M. & Hatten, M.E. (2004) Par6alpha signalling controls glial-guided neuronal migration. *Nat. Neurosci*., **7**, 1195-1203.

Spires, T.L., Molnár, Z., Kind, P.C., Cordery, P.M., Upton, A.L., Blakemore, C. & Hannan, A.J. (2005) Activity-dependent regulation of synapse and dendritic spine morphology in developing barrel cortex requires phospholipase C-beta1 signalling. *Cereb. Cortex*, **15**, 385-393.

Storbeck, C.J., Wagner, S., O'Reilly, P., McKay, M., Parks, R.J., Westphal, H. & Sabourin, L.A. (2009) The Ldb1 and Ldb2 transcriptional cofactors interact with the Ste20-like kinase SLK and regulate cell migration. *Mol. Biol. Cell*., **20**, 4174-4182.

Tanaka, S., Ishii, K., Kasai, K., Yoon, S, O. & Saeki Y. (2007) Neural expression of G protein-coupled receptors GPR3, GPR6, and GPR12 up-regulates cyclic AMP levels and promotes neurite outgrowth. *J. Biol. Chem*., **282**, 10506-10515.

Tanaka, S.S., Yamaguchi, Y.L., Tsoi, B., Lickert, H. & Tam, P.P. (2005) IFITM/Mil/fragilis family proteins IFITM1 and IFITM3 play distinct roles in mouse primordial germ cell homing and repulsion. *Dev. Cell*., **9**, 745-756.

Tang, X., Jang, S.W., Okada, M., Chan, C.B., Feng, Y., Liu, Y., Luo, S.W., Hong, Y., Rama, N., Xiong, W.C., Mehlen, P. & Ye, K. (2008) Netrin-1 mediates neuronal survival through PIKE-L interaction with the dependence receptor UNC5B. *Nat. Cell. Biol*., **10**, 698-706.

Uesugi, K., Oinuma, I., Katoh, H. & Negishi, M. (2009) Different requirement for Rnd GTPases of R-Ras GAP activity of Plexin-C1 and Plexin-D1. *J. Biol. Chem*. **284**, 6743-6751.

Uhrig, M., Brechlin, P., Jahn, O., Knyazev, Y., Weninger, A., Busia, L., Honarnejad, K., Otto, M. & Hartmann, T. (2008) Upregulation of CRABP1 in human neuroblastoma cells overproducing the Alzheimer-typical Abeta42 reduces their differentiation potential. *B. M.C. Med*., **16**, 38.

Wallén, A., Zetterström, R.H., Solomin, L., Arvidsson, M., Olson, L. & Perlmann, T. (1999) Fate of mesencephalic AHD2-expressing dopamine progenitor cells in NURR1 mutant mice. *Exp. Cell. Res*., **253**, 737-746.

Ward, G.R., Franklin, S.O., Gerald, T.M., Dempsey, K.T., Clodfelter, D.E. Jr., Krissinger, D.J., Patel, K.M., Vrana, K.E. & Howlett, A.C. (2007) Glucocorticoids plus opioids up-regulate genes that influence neuronal function. *Cell. Mol. Neurobiol*., **27**, 651-660.

Wu, Y., Sheng, W., Chen, L., Dong, H., Lee, V., Lu, F., Wong, C.S., Lu, W.Y. & Yang, B.B. (2004) Versican V1 isoform induces neuronal differentiation and promotes neurite outgrowth. *Mol. Biol. Cell*., **15**, 2093-2104.

Wu, Y., Arai, A.C., Rumbaugh, G., Srivastava, A.K., Turner, G., Hayashi, T., Suzuki, E., Jiang, Y., Zhang, L., Rodriguez, J., Boyle, J., Tarpey, P., Raymond, F.L., Nevelsteen, J., Froyen, G., Stratton, M., Futreal, A., Gecz, J., Stevenson, R., Schwartz, C.E., Valle, D., Huganir, R.L. & Wang, T. (2007) Mutations in ionotropic AMPA receptor 3 alter channel properties and are associated with moderate cognitive impairment in humans. *Proc. Natl. Acad. Sci. U. S. A*. **104**, 18163-18168.

Xiao, M., Xu, L., Laezza, F., Yamada, K., Feng, S. & Ornitz, D.M. (2007) Impaired hippocampal synaptic transmission and plasticity in mice lacking fibroblast growth factor 14. *Mol. Cell. Neurosci*., **34**, 366-377.

Xu, N., Bhagavath, B., Kim, H.G., Halvorson, L., Podolsky, R.S., Chorich, L.P., Prasad, P., Xiong, W.C., Cameron, R.S. & Layman, L.C. (2010) NELF is a nuclear protein involved in hypothalamic GnRH neuronal migration. *Mol. Cell. Endocrinol*., **319**, 47-55.

Yamada, T., Sakisaka, T., Hisata, S., Baba, T. & Takai, Y. (2005) RA-RhoGAP, Rap-activated Rho GTPase-activating protein implicated in neurite outgrowth through Rho. *J. Biol. Chem*. **280**, 33026-33034.

Yamashita, N., Kosaka, K., Ilg, E.C., Schäfer, B.W., Heizmann, C.W. & Kosaka, T. (1997) Selective association of S100A6 (calcyclin)-immunoreactive astrocytes with the tangential migration pathway of subventricular zone cells in the rat. *Brain Res*., **78**, 388-392.

Yoon, S.Y., Choi, J.E., Huh, J.W., Hwang, O., Lee, H.S., Hong, H.N. & Kim, D. (2005) Monastrol, a selective inhibitor of the mitotic kinesin Eg5, induces a distinctive growth profile of dendrites and axons in primary cortical neuron cultures. *Cell. Motil. Cytoskeleton*, **60**, 181-190.

Zorina, Y., Iyengar, R. & Bromberg, K.D. (2010) Cannabinoid 1 receptor and interleukin-6 receptor together induce integration of protein kinase and transcription factor signalling to trigger neurite outgrowth. *J. Biol. Chem*., **285**, 1358-1370.
